# T helper cell 17/regulatory T cell balance regulates ulcerative colitis and the therapeutic role of natural plant components: a review

**DOI:** 10.3389/fmed.2024.1502849

**Published:** 2025-03-24

**Authors:** Da Zhao, Anqi Ge, Cong Yan, Xingci Liu, Kailin Yang, Yexing Yan, Moujia Hao, Junpeng Chen, Pawan Daga, Charles C. Dai, Changping Li, Hui Cao

**Affiliations:** ^1^The First Hospital of Hunan University of Chinese Medicine, Changsha, China; ^2^Department of Urology, The Affiliated Children’s Hospital of Xiangya School of Medicine, Central South University (Hunan Children’s Hospital), Changsha, China; ^3^Department of Psychology, Daqing Hospital of Traditional Chinese Medicine, Daqing, China; ^4^Tong Jiecheng Studio, Hunan University of Science and Technology, Xiangtan, China; ^5^Department of Physiology, University of Louisville School of Medicine, Louisville, KY, United States; ^6^Pediatric Research Institute, Department of Pediatrics, University of Louisville School of Medicine, Louisville, KY, United States; ^7^Center for Cardiometabolic Science, Division of Environmental Medicine, Christina Lee Brown Envirome Insttitute, University of Louisville, Louisville, KY, United States; ^8^Department of Internal Medicine, University of Louisville, Louisville, KY, United States; ^9^Department of Oral and Maxillofacial Surgery, School of Dentistry, University of Maryland Baltimore, Baltimore, MD, United States; ^10^Fischell Department of Bioengineering, A. James Clark School of Engineering, University of Maryland, James Clark Hall, College Park, MD, United States; ^11^School of Mechanical Engineering and Automation, Fuyao University of Science and Technology, Fuzhou, China

**Keywords:** Th17/Treg balance, ulcerative colitis, immune inflammation, natural plant components, inflammatory disease

## Abstract

Ulcerative colitis (UC) is a chronic relapsing inflammatory disease characterized by progressive mucosal damage. The incidence rate of UC is rising rapidly, which makes the burden of medical resources aggravated. In UC, due to various pathogenic factors such as mucosal immune system disorders, gene mutations and environmental factors disrupting the mucosal barrier function, the midgut pathogenic bacteria and exogenous antigens translocate into the lamina propria, thereby aggravating the inflammatory response and further damages the mucosal barrier. During the progression of UC, Th17 populations that cause inflammation generally increase, while Tregs that suppress Th17 activity decrease. Among them, Th17 mediates immune response, Treg mediates immunosuppression, and the coordinated balance of the two plays a key role in the inflammation and immune process of UC. Natural plant components can regulate biological processes such as immune inflammation from multiple levels of proinflammatory cytokines and signaling pathways. These characteristics have unique advantages and broad prospects in the treatment of UC. In immunomodulation, there is substantial clinical and experimental evidence for the modulatory role of natural plant products in restoring balance between Th17/Treg disturbances in UC. This review summarizes the previous studies on the regulation of Th17/Treg balance in UC by natural plant active ingredients, extracts, and traditional Chinese medicine prescriptions, and provides new evidence for the development and design of lead compounds and natural new drugs for the regulation of Th17/Treg balance in the future, and then provides ideas and evidence for future clinical intervention in the treatment of UC immune disorders and clinical trials.

## 1 Introduction

Ulcerative colitis (UC) is a chronic idiopathic intestinal inflammatory disease (IBD) with multiple etiologies and unknown pathological mechanism (1). The lesion starts from the rectum and usually extends in a continuous manner to part of the colon or even the entire colon, and the inflammation is mainly limited to the mucosal surface. Bloody diarrhea and abdominal pain are characteristic symptoms of UC (2, 3). The clinical course is unpredictable, with periods of attack and remission alternating. The global prevalence of UC is estimated to be 5 million cases in 2023, and the incidence is increasing worldwide, with similar incidence in men and women (4, 5). Furthermore, the rise in IBD cases in regions like China and India has been notable, marking a transition from an uncommon pathology to a prevalent health concern (5). Individuals grappling with persistent and/or widespread UC face heightened susceptibility to colorectal cancer (CRC) when contrasted with the broader populace, given the intricate nature of the disease, its prolonged course, and inherent cancer predisposition (6). A meta-analysis showed that the cumulative probability of developing colorectal cancer among all patients with UC was 2% at 10 years, 8% at 20 years, and 18% at 30 years (7, 8).

The pathological factors causing UC mainly include heredity, mental psychology, external environment, and immune factors (3, 9). At present, it is believed that immune imbalance is the key factor of recurrent UC, protracted and difficult to heal (10). The research on the immune mechanism of UC has changed from the balance between Th1/Th2 in the early stage to the balance of Th17/Treg in recent years (11). Studies have shown that intestinal microbiota and their metabolites can affect the immune balance of Th17/Treg, and induce the body to produce an adaptive immune response under pathological conditions (12). The dysregulation between Th17 and Treg cells plays a pivotal role in the pathogenesis of UC. Particularly, Th17 cells are implicated in the initiation and advancement of diverse autoimmune conditions, including IBD (13). Activation of Th17 cells is facilitated by IL-23, while the transcription factor RORγt governs the differentiation and functional modifications of these cells. Th17 cells primarily secrete pro-inflammatory cytokines like IL-17 and IL-21. On the other hand, Foxp3 acts as a crucial transcription factor for Treg cells, regulating their development and functionality. Treg cells primarily release anti-inflammatory agents such as IL-10 and TGF-*β*, collectively orchestrating immune modulation (14, 15). Any disruption in the equilibrium between Th17 and Treg cells prompts alterations in their respective cytokine profiles, consequently triggering a cascade of immune responses and inflammatory reactions. Excessive immune reactions culminate in tissue injury and are intricately involved in both the onset and progression of UC (16).

At present, the clinical treatment of UC is mainly based on 5-amino acid salicylic acid maintenance therapy, glucocorticoids, immunosuppressants, etc. to control the acute attack. But its treatment time is long, easy to relapse, seriously reduces the quality of life of patients, and increases psychological and economic challenges (17–19). In recent years, the advantages of natural plant compounds in the treatment of UC have gradually become prominent, so the molecular mechanism of anti-inflammatory and anti-immune properties of natural plant compounds has been studied (20). In addition, there are many reports on the mechanism of natural plant components in regulating Th17/Treg cells (21). Our previous work also studied the molecular biological process of multi-component compounds intervening in the anti-inflammatory and anti-immune process of UC (22, 23). In order to provide lead compounds or natural plant components of parent nucleus structure for future drug development targeting the anti-inflammatory and anti-immune process of Th17/Treg cells, this review will not only summarize the research progress of the mechanism of Th17/Treg cells in UC, but also summarize the drugs of natural plant components regulating Th17/Treg cells.

## 2 Literature search methods

### 2.1 Search strategy

The reviewers conducted a thorough literature search in databases including China National Knowledge Infrastructure (CNKI), Medline Complete, Web of Science, Wanfang Database, PubMed, Sinomed, Embase, VIP Database the Cochrane Library and ClinicalTrials.gov. The search covered the period from the inception of the databases until February 1, 2024. For instance, the search strategies for PubMed and Embase are outlined in Supplementary Table S1.

### 2.2 Search criteria

Inclusion criteria: (1) Participants: Patients diagnosed with UC, UC animal models, or UC-related cell models, regardless of type or race, must adhere to medical ethics. (2) Intervention: Natural plant components as intervention methods, with no restrictions on dosage type, route of administration, or preparation methods, including natural plant ingredient monomers, components, or active ingredients, as well as traditional Chinese medicine (TCM) prescriptions, but must be in accordance with medical ethics. (3) Outcomes: Including any changes in Th17/Treg. (4) Study Design: Basic or clinical trials.

Exclusion criteria: (1) Studies not adhering to medical ethics; (2) Retracted studies.

### 2.3 Literature screening and data management

**Figure 1:**
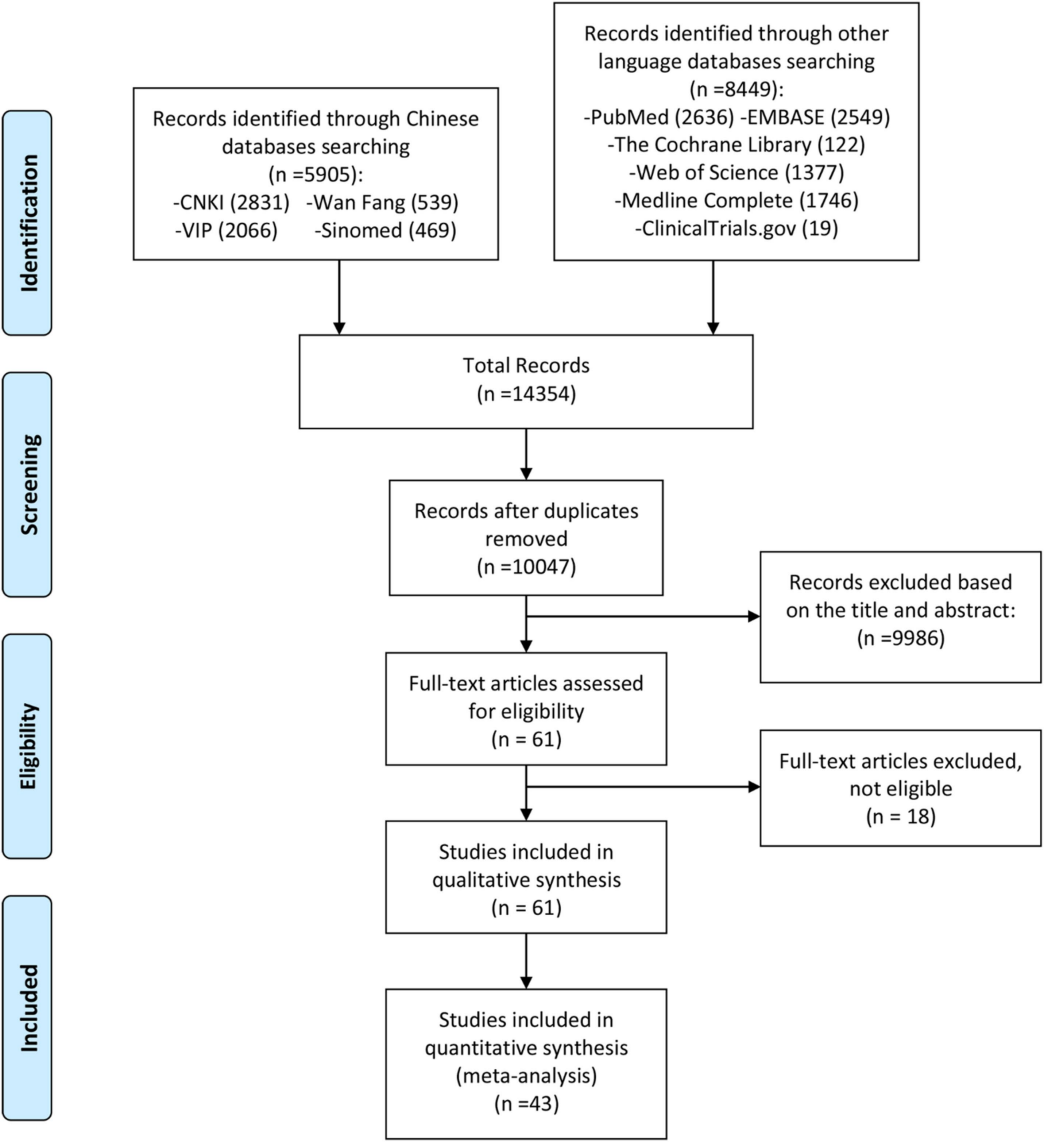
Literature screening process.

The reviewers initially conducted a preliminary search based on the search formula outlined in Supplementary Table S1. After removing duplicate publications from the preliminary search, two reviewers independently screened publications by reviewing titles, abstracts, and keywords according to the following principles: advancement of a publication to the next screening stage required agreement from only one reviewer, whereas exclusion of a publication necessitated agreement from both reviewers. Subsequently, the two reviewers further screened the articles based on search criteria, including articles that met the criteria (see Figure 1). The included articles are presented in Tables 1, 2.

**Table 1:** Summary of the mechanism of phytochemicals in the treatment of UC.

Phytochemicals	Species	Model/disease	Functions	Reference
Total glycosides of paeony	*Rattus norvegicus*	TNBS-induced colitis	Reduce the levels of Th17-related cytokines IL-17 and TNF-α and increase the levels of Treg-related cytokines TNF-β and IL-10	(103)
Paeoniflorin	*Rattus norvegicus*	TNBS-induced colitis	Regulated the Th17/Treg balance and inhibited the activation of NF-κB signaling pathway	(104)
	*Rattus norvegicus*	DSS-induced colitis	Decrease the expression of IL-17, reduce the secretion of IL-17, TNF-α and ICAM, and up-regulate the expression of autophagy-related factors LC3BII and Beclin1	(106)
Neomangiferin	*Mus musculus*	TNBS-induced colitis	Inhibited TGF-β/IL-6-induced differentiation of splenic T cells into Th17 cells, increased GM-CSF-induced differentiation of splenic T cells into Treg cells, restored the balance between Th17/Treg cells by inhibiting the expression of IL-17 and RORγt and inducing the expression of IL-10 and FOXP3	(115)
Mangiferin	*Mus musculus*	TNBS-induced colitis	Suppressed the maturation of Th17 cells and the expression of IL-17, enhanced the generation of Treg cells and the expression of IL-10; impeded the development of splenocytes into Th7 cells while boosting differentiation into Treg cells in experimental settings, dampened the activity of RORγt and IL-17 as well as the activation of STAT3 in splenocytes, and sparked the upregulation of Foxp3 and IL-10 coupled with the activation of STAT5.	(117)
Temosaponin AIII and its metabolite sarsasapogenin	*Mus musculus*	TNBS-induced colitis	Suppressed the transformation of CD4 T cells from the spleen into Th17 cells within a laboratory setting, hindered the TLR4-NF-κB/MAPK signaling pathway, and reinstated equilibrium between Th17 and Treg cell populations.	(119)
Oleanolic acid	*Mus musculus*	DSS-induced colitis	Inhibited Th17 cell differentiation, downregulated the expression of RORγt and IL-17 in the colon and in the lamina propria of Treg cell differentiation, and increased the expression of Foxp3 and IL-10	(125)
Ocotillol and Majonoside R2	*Mus musculus*	TNBS-induced colitis	Inhibited the expression of TNF-α and IL-1β, and the activation of NF-κB and MAPK; inhibited the differentiation of Th17 cells in the colonic lamina propria, as well as the expression of T-bet, RORγt, IL-17, and IL-23, and increased Treg cell differentiation and the expression of Foxp3 and IL-10	(129)
Madecassoside and Madecassic acid	*Mus musculus*	DSS-induced colitis	Modulated PPARγ/AMPK/ACC1 pathway to restore Th17/Treg balance to improve UC	(134)
Curcumin	*Mus musculus*	DSS-induced colitis	Regulated the balance of Treg/Th17 to increase the anti-inflammatory cytokine IL-10, while reducing the pro-inflammatory cytokines IL-23, IL-17 and IL-6	(140)
Baicalin	*Rattus norvegicus*	TNBS-induced colitis	Downregulated the number of Th17 cells as well as the levels of Th17-related cytokines (IL-17 and IL-6) and RORγt; increased the number of Treg, and the levels of Treg-associated cytokines TGF-β and IL-10 and FOXP3	(142)
Isoliquiritigenin and Naringinin	*Mus musculus*	DSS-induced colitis	Promoted Treg cell induction	(147)
Berberine	*Mus musculus*	DSS-induced colitis	Improved the balance of Treg/Th17 in and reduced the diversity of intestinal microbiota	(149)
Parthenolide	*Mus musculus*	DSS-induced colitis	Upregulated the Treg cells and downregulate the proportion of colonic Th17 cells and improved Treg/Th17 balance to maintain intestinal homeostasis.	(152)
Polydatin	*Mus musculus*	TNBS-induced colitis and DSS-induced colitis	Reduced the proportion of Th17 cells	(154)
Resveratrol	*Mus musculus*	DSS-induced colitis	Regulated Treg/Th17 rebalance, increased TGF-β1 and IL-10 levels, reduced IL-6 and IL-17 levels, and inhibited hypoxia-mTOR-HIF-1α-Th17 and IL-6-STAT3-HIF-1α-Th17 pathways	(159, 160)
Dihydromyricelin	*Mus musculus*	DSS-induced colitis	Restored the balance of Treg/Th17 in peripheral blood and reduce the expression of MMP9 in colon tissue	(162)
Daphnetine	*Mus musculus*	DSS-induced colitis	Enhanced Treg development and reduced h17 cell differentiation of pro-inflammatory T cells	(168)
Stigmasterol	*Mus musculus*	DSS-induced colitis	Restored the balance of Treg/Th17 cells through butyrate-mediated activation of PPARγ	(173)
Tangeretin	*Mus musculus*	TNBS-induced colitis	Inhibited the differentiation of Th1 and Th17 cells and the expression of T-bet, RORγt, interferon-γ, IL-12, IL-17 and TNF-α; increased the differentiation of Treg and the expression of Foxp3 and IL-10.	(182)
Andrographolide	*Mus musculus*	TNBS-induced colitis	Inhibited CD4+ T cell infiltration and differentiation of Th1 (CD4+ IFN-γ+) and Th17 (CD4+ IL17A+).	(187)
Astragaloside IV	*Mus musculus*	DSS-induced colitis	Inhibited the inflammatory response in mice with colitis and reduce the ratio of peripheral blood Th17 cells/Treg	(193)
Astragalus polysaccharide	*Mus musculus*	DSS-induced colitis	Regulated the differentiation of Tfh subsets, up-regulated Tfh10 and Tfr, and down-regulated Tfh1, Tfh17 and Tfh21; up-regulated the levels of Treg cells and their related nuclear transcription factor Foxp3 and cytokine IL-10.	(198)
Icariin	*Mus musculus*	DSS-induced colitis	Inhibited the phosphorylation of STAT1 and STAT3, which are key transcription factors for Th1 and Th17, respectively, in CD4+ T cells	(202)
Epigallocatechin-3-gallate	*Mus musculus*	DSS-induced colitis	Decreased the release of IL-6 and IL-17, regulated the ratio of Treg/Th17 in the spleen of mice, while increased the plasma levels of IL-10 and TGF-β1, and decreased the expression of HIF-1α and STAT3 proteins in the colon	(39)
Arctigenin	*Mus musculus*	DSS-induced colitis	Inhibited mTORC1 activation and thereby preventing Th17 cell differentiation and the development of colitis	(209)
3,3′-Diindolylmethane	*Mus musculus*	Oxazolone-induced colitis	Decreased Th2/Th17 cells and increased Treg	(214)
Artemisinin	*Mus musculus*	TNBS-induced colitis	Promoted HO-1 production in vitro and in vivo, accompanied by CD4+ T cell apoptosis and restoration of Th/Treg balance	(219)
Juglone	*Mus musculus*	DSS-induced colitis	Inhibited Th17 development and increased Treg generation, which is beneficial to Th17/Treg balance	(224)
Nuciferine	*Mus musculus*	DSS-induced colitis	Improved Th1/Th2 and Treg/Th17 balance	(230)

**Table 2:** Summary of the mechanism of TCM prescriptions in the treatment of UC.

TCM prescriptions	Species	Model/disease	Functions	Reference
Kuijieling	*Rattus norvegicus*	TNBS-induced colitis	Increased blood TGF-β1, IL-2, IL-10 and colon Foxp3, STAT5, IL-2, decreased blood IL-6, IL-23, IL-17, IL-21 and colon RORγt, STAT3, IL-23, IL-17, IL-21; promoted Treg cells and suppressing Th17 cells	(236)
Baitouwen Tang	*Homo sapiens*	UC	Increased the proportion of Treg cells and reduced the proportion of Th17 cells in patients	(240)
Fuzi Lizhong Tang	*Rattus norvegicus*	TNBS-induced colitis	Upregulated the levels of Treg-related factors IL-10, TGF-β1, Foxp3, and STAT5	(244)
Qingchang Wenzhong Tang	*Mus musculus*	DSS-induced colitis	Inhibited the IL-6-STAT3 pathway and inhibited the differentiation of Th17 lymphocytes	(247)
Fufang Kushen Tang	*Mus musculus*	DSS-induced colitis	Improved the symptoms and pathological damage of colitis mice and affected immune function by regulating the balance of Th17/Treg cells	(253)
Dahuang Mudan Tang	*Mus musculus*	DSS-induced colitis	Improved the ratio of Th17 cells and Treg cells in mesenteric lymph nodes, and decreased the levels of IL-6, TNF-α, IFNγ, IL-10, IL-17A, IL-21, and IL-22	(255)
Gegen Qinlian Tang	*Mus musculus*	DSS-induced colitis	Restored Treg and Th17 cell homeostasis in colon tissue by inhibiting IL-6/JAK2/STAT3 signaling	(259, 260)
Shenling Baizhu San	*Rattus norvegicus*	TNBS-induced colitis	Regulated the expression level of RORγt/FoxP3 and correcting the immune imbalance of Th17/Treg	(262)
*Panax ginseng* C. A. Mey. Extracts	*Mus musculus*	TNBS-induced colitis	Inhibited the binding of LPS to TLR4 on macrophages and restored Th17/Treg imbalance.	(263)
Shaoyao Tang	*Rattus norvegicus*	TNBS-induced colitis	Regulated the balance of Treg/Th17 by inhibiting HIF-1α	(22)
	*Homo sapiens*	UC	Promoted the recovery of intestinal mucosa, and improved the quality of life of patients by regulating the balance of Th17/Treg cells	(276)

## 3 Pathophysiological changes of UC

### 3.1 Microbiota

The gut microbiota represents a complex assembly of microorganisms that inhabit the gastrointestinal tract. Alterations in the gut microbiota can impact populations of intestinal mucosal immune cells, including macrophages and dendritic cells, which can activate T and B cells through antigen presentation (24).

The pathogenesis of UC is strongly associated with disruptions in the intestinal microbiota. Normally, beneficial and pathogenic bacteria in the gut tract engage in reciprocal regulation to sustain the dynamic equilibrium of the immune-inflammatory system (25). Perturbations in the intestinal flora can prompt aberrant immune and inflammatory responses within the intestinal mucosa, leading to damage to the mucosal barrier, heightened epithelial permeability, bacterial and endotoxin accumulation in the lamina propria, anomalous immune responses, excessive inflammatory factor release, and the onset of UC (13, 26). Simultaneously, mucosal barrier impairment can alter pathogenic microorganism composition, exacerbating intestinal microbiota imbalance, thereby creating a detrimental feedback loop (27). Consequently, compromised intestinal mucosal barrier function stands as a pivotal pathological mechanism in UC pathogenesis.

Numerous studies have emphasized the pivotal role of balanced gut microbiota in upholding intestinal mucosal barrier integrity (28). Conversely, disruptions in the microbiota can disrupt normal mucosal barrier function, impact mucosal immune and inflammatory responses, and thus drive UC progression (29). Research indicates that interventions aimed at regulating the intestinal microbiota, such as probiotics, prebiotics, fecal transplantation, among others, can restore microbiota homeostasis, mend mucosal barrier integrity, and inhibit UC advancement, offering a promising avenue for UC treatment (30–32).

### 3.2 Mucosal immunity

Mucosal immunity encompasses the entire internal surface of the body and serves as the primary defense mechanism against infections. The intestinal mucosal barrier, governed by the equilibrium of intestinal microbiota and its molecular sealing capacity, forms the foundation of mucosal immunity. Pathogen-associated molecular patterns released from the surface of intestinal bacteria bind to Toll-like receptors (TLR) on immune cell membranes and NOD-like receptors (NLR) in the intracellular environment, activating nuclear factor-kappa B (NF-κB) and transcription factor AP-1. This activation leads to the transcription of inflammatory genes, the release of inflammatory mediators, and the recruitment and activation of inflammatory cells, initiating the onset of UC (33). Inflammation generates an overabundance of reactive oxygen species, causing crypt abscesses and tissue damage, hallmarks of typical UC ulcers (34). Dendritic cells (DCs) facilitate and engage adaptive immunity through antigen presentation and cytokine secretion, activating CD4+ T effector cells, guiding and stimulating effector T cells, and subsequently inducing B cells to produce anti-inflammatory and mucosal protective secretory immunoglobulin A (SIgA). Ultimately, the subcellular imbalance between pro-inflammatory Th1, Th17 responses, and anti-inflammatory effects tips in favor of inflammation. The resulting mucosal damage from inflammation perpetuates ongoing colonic inflammation and ulceration (35, 36).

### 3.3 The mechanism of Th cells in UC

Regulatory T cells (Treg cells) are a subset of CD4+ T cells suppressing immune responses. Although Treg cells constitute less than 10% of CD4+ T cells, they are critical in preventing autoimmune disease and chronic inflammatory diseases, including gut inflammation (37, 38). Utilizing a murine model of UC, augmenting IL-10 and TGF-*β* production significantly alleviates diarrheal symptoms, illustrating the ability of Treg cells to dampen intestinal inflammatory cascades and exaggerated responses by modulating the secretion of anti-inflammatory factors like IL-10 and TGF-β, thus ameliorating UC’s clinical manifestations (39). Flow cytometry results demonstrated a reduction in the number of peripheral blood Treg cells in UC mice, aligning with the notion that Treg cells possess the capability to regulate intestinal inflammation (40, 41). Evidence also suggests that disruptions in intestinal tolerance resulting in deficiencies in Treg cell number and function may foster UC onset and progression (41). Clinical and animal studies emphasize Treg cells’ critical role in preventing gut inflammation, and their deficiencies may contribute to UC (37). Despite growing interest in Treg cells, research on their anomalies in human UC remains limited. Treg cells are recognized for producing essential cytokines like IL-10 and TGF-*β*, with IL-10 specifically regulating immune responses. By modulating the expression and release of pro-inflammatory molecules such as IL-1β, IL-6, and TNF-*α*, IL-10 effectively impedes antigen presentation, thereby restraining T cell-mediated immune responses (42). Studies involving IL-10 deficient mice revealed their susceptibility to developing colitis, mirroring human UC pathogenesis and highlighting IL-10’s crucial role in mitigating UC progression. Notably, administering IL-10-enriched bifidobacteria orally improved UC symptoms in mice, underscoring IL-10’s significance in UC management (43). Tao et al. observed reduced levels of peripheral blood Treg cells and IL-10 in UC-afflicted mice compared to controls in their research (44). Elevating serum and mucosal IL-10 levels, coupled with enhancing peripheral Treg cell counts, notably ameliorated colonic damage in UC mice, demonstrating the effective collaboration between IL-10 and Treg cells in reducing colon damage in UC mice (44). Furthermore, Treg cells also secrete TGF-*β*, a multifunctional cytokine with potent regulatory effects on inflammation, angiogenesis, immune response inhibition, and even tumor growth, invasion, and metastasis (45). Xu et al. discovered that Kuijie Granules could alleviate various UC symptoms by reducing TGF-*β* expression in observations of this medicine’s therapeutic effects on UC patients (46). Another researcher established a UC mouse model, noting a significant rise in TGF-β levels during active UC phases, positively correlating with disease severity, suggesting a role for TGF-*β* in promoting UC onset and progression (47).

## 4 Mechanism of Th17/Treg balance in UC

Upon encountering antigen signals, naïve CD4+ T lymphocytes are stimulated and activated through co-stimulatory signals and molecules, leading to differentiation into various T lymphocyte subsets under specific conditions. These diverse cell types include T17 and Treg cells (48). Naïve CD4+ T cells transition into Treg cells under the influence of TGF-*β*, while differentiation into Th17 cells is facilitated by the combined effects of TGF-β and IL-6. Th17 and Treg cells have a reciprocal relationship during differentiation, mutually suppressing each other’s functions to maintain immune homeostasis within the body. Perturbation of this balance can result in the development of autoimmune disorders, including UC (49). Several studies have underscored the crucial role of the Th17/Treg balance in the inflammatory and immune processes of UC. An imbalance between Th17 and Treg cells leads to elevated serum levels of Th17-associated cytokines (such as IL-6, IL17A, and IL-17F) alongside diminished levels of Treg-related cytokines (like IL-10, IL-2, and TGF-*β*) (50, 51). Additionally, these cytokines are likely to act synergistically to induce damage to colonic epithelial cells, thereby contributing to local tissue inflammation. Restoring the immune balance between Th17 and Treg cells holds promise as a novel therapeutic avenue for managing UC (52). Gong et al. discovered that the upregulation of Th17 cells in UC patients correlates with increased serum IL-17 levels, while a decrease in Treg cells leads to reduced TGF-*β* levels, activating autoreactive T cells and decreasing immunosuppressive factors, thereby exacerbating colonic mucosal inflammation. This suggests the presence of a clear Th17/Treg imbalance in UC patients, which may play a pivotal role in UC development (53). Ma et al. observed an imbalance in the Th17/Treg cell ratio in UC patients, where a decrease in the Treg cell ratio was associated with serum indicators of inflammatory activity, indicating a link between Th17/Treg cell imbalance, UC disease activity, and inflammation (54). Luo et al. noted a significant decrease in the number of Treg cells in UC patients’ serum and colon tissue, accompanied by a considerable increase in Th17 cells, indicating a notable Treg/Th17 cell imbalance in UC patients, positively correlated with intestinal inflammation (55). The factors influencing UC pathogenesis are not solely the reduction of Treg cells or the increase of Th17 cells but rather the interplay between Treg and Th17 cells leading to immune imbalance (56). An evident imbalance exists in the Th17/Treg cell ratio in UC, potentially being a key aspect of UC pathogenesis. Throughout the progression of UC, inflammatory Th17 populations typically increase, while Tregs, responsible for suppressing Th17 activity, decrease. Among these, Th17 mediates immune responses, while Treg mediates immunosuppression, with the balanced interplay between the two being crucial in the inflammatory and immune processes of UC. Therefore, regulating the Th17/Treg cell balance to enhance Treg cells and suppress Th17 cells may play a significant role in protecting the intestinal mucosa in UC.

## 5 UC-related signaling pathways regulating Th17/Treg balance

Currently, extensive research indicates that the modulation of the Th17/Treg equilibrium can effectively regulate UC. This modulation, in essence, establishes an immune regulatory framework by integrating diverse cytokines and transcription factors within intricate signaling pathways both upstream and downstream.

### 5.1 IL-6/JAK/STAT3 signaling pathway

Cellular responses to IL-6 are orchestrated by a receptor complex comprising IL-6Rα and glycoprotein 130 (gp130) (57). Within the JAK–STAT signaling cascade, two JAK isoforms function as autophosphorylated homodimers or heterodimers, facilitating the recruitment and phosphorylation of various signaling molecules, including members of the STAT protein family, which are crucial for DNA-binding activities (58). Comprising four intracellular tyrosine protein kinases, the JAK family (JAK1-45I, JAK2, JAK3, and TYK2) activates STAT proteins—such as STAT1-4, STAT5a, STAT5b, and STAT6 (31)—sparking diverse downstream cellular responses (59). In the IL-6/JAK/STAT3 signaling axis, IL-6 binding to glycoprotein 130 receptors (gp130R) triggers tyrosine phosphorylation and activates JAKs linked to gp130R, promoting cytoplasmic STAT3 transcription factor activation (60). Phosphorylated STAT3 dimers are subsequently translocated into the nucleus to regulate gene transcription, participating in inflammatory responses (61). Activation through IL-6R/gp130 leads to a pro-inflammatory function for STAT3, contrasting with its anti-inflammatory role when stimulated by IL-10R (62). In gut-associated lymphoid tissues, dendritic cells and other antigen-presenting cells initiate antigen-specific immune responses, which are critical in determining B cell activation and the early differentiation of T helper cells through JAK–STAT-mediated cytokine-receptor interactions (63). These processes lead to the differentiation and expansion of TH1, TH2, TH9, and TH17 effector cells within the intestinal mucosa. In turn, TH cell subtypes regulate other immune cells, including CD8+ cytotoxic cells, regulatory T cells (Treg), macrophages, and dendritic cells, through cytokine-receptor interactions and JAK–STAT signaling pathways (60). Consequently, JAK inhibitors have the potential to broadly impact immune mechanisms underlying IBD, affecting inflammatory responses, the intestinal epithelial barrier, and fibrosis (60).

### 5.2 TLR4/MyD88/NF-KB signaling pathway

TLRs are a type I transmembrane receptor superfamily (64), which play an important role in the innate immune response against pathogenic microorganisms or tissue damage. After TLR is stimulated, it activates the intracellular cascade reaction and then transmits the stimulating signal to the lower signaling protein NF-KB coupled with MyD88 (65). Activated NF-κB can participate in the transcriptional regulation of various target genes, resulting in the release of various inflammatory factors (such as TNF-*α*, interferon-*γ*, IL-1β, IL-6) and antimicrobial peptides (66, 67), resulting in intestinal tissue damage and IBD (67, 68). In addition, the differentiation of Th17 and Treg is also regulated by NF-κB. NF-κB comprises a dimer of five proteins of the Rel transcription factor family: p105/p50, p100/p52, RelA (p65), RelB, and c-Rel, which shares N-terminal homology with the v-Rel oncogene (69). iTreg generation in c-Rel-deficient CD4+ T cells was severely hindered, and was associated with a decreased number of Foxp3* T cells *in vivo* (53). c-Rel is highly activated in the thymus regulatory T precursor cells (pre-tTreg) with high expression of CCR7, which initiates the transcription of Treg-related coding genes and promotes the differentiation of pre-tTreg into thymic regulatory T cells (tTreg) (70). Ablation of NF-KBc-Rel can specifically impair the generation and maintenance of activated regulatory T cells (aTreg) (71). In addition, c-Rel and p65 can drive Treg development by promoting the formation of Foxp3-specific enhancers (72). Rorg/Rorc are direct targets of the transcription factor p65 in Th17 cells. Therefore, when c-Rel or p65 is deficient, the expression of RORyt mRNA is reduced, the differentiation of CD4 + T to Th17 is weakened, and the expression of IL-17A/IL-17F of Th17 is reduced (73).

### 5.3 mTOR signaling pathway

The mammalian target of rapamycin (mTOR) serves as a pivotal regulator of growth and development, modulated by an array of extracellular and intracellular factors like amino acids, energy levels, and hormonal cues (74). Notably, during inflammatory conditions, the hyperactivation of the PI3K/Akt–mTOR signaling pathway emerges as a significant player in the context of UC-associated carcinogenesis (75). In a study by Chi et al., heightened expression of the key transcription factor RORα, which oversees Th17 cell regulation, was notably observed in individuals with active UC, particularly in those unresponsive to TNF antagonist therapy. Utilizing a mouse model of T cell-triggered enteritis, they demonstrated that ablation of RORA in CD4 T cells substantially mitigated the severity of enteritis induced by T cells. RORα facilitated the migration of mesenteric lymph node T cells to the gut while impeding the apoptosis of resident intestinal T cells, thereby fostering T cell infiltration in the intestine. Furthermore, RORα prompted CD4 T cells to release potent cytokines like IL-17 and TNFα, thereby enhancing their pathogenicity. Through ChIP-seq and RNA-seq analyses, the researchers unearthed that RORα bolstered the activation of the mTORC1 signaling pathway, with the deletion of the mTORC1-specific component Rptor in T cells significantly curbing T cell pathogenicity in enteritis. Moreover, abnormal mTORC1 signaling was evident in active UC patients, showing a positive correlation with RORα expression levels. In essence, the pivotal interplay between the RORα-mTORC1 axis in modulating CD4 T cell enteritis pathogenicity may unveil a promising therapeutic target for managing IBD (76).

The mTOR signaling pathway plays a dual role in not only repairing intestinal mucosal damage but also sustaining the regular metabolism of intestinal epithelial cells. Often dysregulated in various conditions like tumors (75), leukemia (77), vascular and skin growth disorders (78), this pathway is intricately linked to the development of gastrointestinal malignancies stemming from prolonged IBD affliction. Within effector CD4+ T cells, mTOR facilitates the differentiation of Th1, Th2, and particularly Th17 cells, crucial for both *in vitro* and *in vivo* contexts, as T cells deficient in mTOR fail to mature into Th17 cells (79). In cell culture settings, microRNAs (miRNAs) exert control over mTOR signaling, prompting either the induction or suppression of autophagy in intestinal cells, releasing either anti-inflammatory or pro-inflammatory factors, respectively (80). By inhibiting mTOR-induced Foxp3 expression, Rapamycin (RAPA) fosters Treg generation from naïve CD4+ T cells, potentially through the restriction of essential amino acids (EAAs) (81). Evidently, the PI3K-AKT–mTOR axis tightly correlates with the Th17/Treg equilibrium, with this signaling pathway showing promise in regulating IBD via the modulation of Th17/Treg balance.

### 5.4 The mechanism of AhR regulation on Th17/Treg differentiation

Initially identified for its role in modulating the toxicity and immune effects of 2,3,7,8-tetrachlorodibenzo-p-dioxin (TCDD) (82), the aryl hydrocarbon receptor (AhR) exhibits widespread distribution across various bodily tissues and cells. Specifically enriched in select CD4+ T cell subsets such as certain hematopoietic stem cells, bone marrow-derived dendritic cells, Langerhans cells, and Th17 cells, AhR displays diminished expression levels in B lymphocytes and Treg cells (83). AhR has a variety of ligands, which can be divided into endogenous and exogenous. Endogenous sources include 6-formylindolo (3,2-b) carbazole (FICZ), a photochemical product of tryptophan, bilirubin, a metabolite of heme via the liver, and lipoxin A4, a metabolite of arachidonic acid. Exogenous sources include environmental pollutants TCDD and benzopyrene formed during the combustion of organic matter inhaled through the respiratory tract, as well as quercetin, indole-3-carbinol, resveratrol, and curcumin in normal diets. Since different ligands can trigger different results, the biological functions and responses of AhR after binding to ligands with different structures are also different (83).

AhR’s role in regulating the differentiation of Th17 and Treg cells hinges on the activation of AhR ligands, failure of which can perturb the differentiation process of naïve T cells. AhR ligands modulate both innate and adaptive immune responses by engaging with AhR in immune cells (83). Recent investigations on T cell polarization in mammals reveal that T cells can fine-tune their differentiation trajectory through orchestrated expression of pivotal transcription factors and appropriate epigenetic alterations (84). Current studies delving into the mechanism of AhR-mediated regulation of Th17/Treg cell differentiation primarily scrutinize various components such as antigen-presenting cells, cytokines, transcription factors, epigenetic modifications, and other facets within activated T lymphocytes (85). As for the regulation of antigen-presenting cells, dendritic cells (DCs) actively engage in antigen uptake, processing, and presentation vital for immune defense. Renowned as the body’s most efficient antigen-presenting cells (APCs), DCs significantly influence T lymphocyte activation (86). Noteworthy is the pivotal immunomodulatory role played by AhR in DCs (87). Recent research points to indoleamine 2,3-dioxygenase (IDO) production dependency in DCs on AhR expression (88). IDO acts as a crucial rate-limiting enzyme in tryptophan breakdown, converting tryptophan into kynurenine. AhR modulates the metabolism of IDO in DCs, thereby influencing the differentiation and functionality of T lymphocytes. Upon TCDD-induced AhR activation, heightened IDO enzyme activation ensues, amplifying the mRNA expression levels of IDO1 and IDO2 (89). Tryptophan can promote the differentiation of Th17, while kynurenine can accelerate the apoptosis of effector T cells and induce the differentiation of Treg. Activated AhR promotes the expression of IDO in DCs, IDO decomposes tryptophan into kynurenine, and kynurenine further induces naïve T lymphocytes to differentiate into Tregs (90). In terms of regulating cytokine levels, Th17 and Treg depend on different cytokines during differentiation (91). The differentiation of induced regulatory T cells (iTreg) and Th17 cells through the aryl hydrocarbon receptor (AhR) may be contingent upon the presence of IL-6 and TGF-*β*. Lower concentrations of IL-6 and TGF-*β* have the capacity to heighten RORγt expression, thereby fostering Th17 differentiation. Conversely, elevated TGF-β levels can instigate Foxp3+ expression, consequently encouraging iTreg differentiation (73, 92). Research by Kimura and colleagues revealed that the standalone presence of TCDD or FICZ ligands fails to induce differentiation into Th17 and iTreg cells. However, in the presence of IL-6 and TGF-β cytokines, TCDD or FICZ can augment Th17 differentiation and IL-17 secretion (93). Additionally, under the influence of TGF-β cytokine, TCDD or FICZ can elevate Foxp3 expression. Regarding the modulation of transcription factor levels, in the context of inflammatory bowel disease (IBD), the JAK/STAT signaling pathway disrupts the T cell balance, thereby influencing the inflammatory response (94). IL-6-mediated STAT3 signaling pathway can upregulate Th17 differentiation. Chaudhry et al. believed that the activation of STAT3 in Treg cells could enable Treg to inhibit Th17 inflammatory response by increasing the expression of inhibitory cell molecules and chemokine receptors, and the loss of STAT3 in Treg cells could induce colitis (95). In addition to stimulating the STAT3 signaling pathway, Th17 differentiation can also inhibit Th17 differentiation through IFN-*γ* or IL-27-mediated STAT1 signaling pathway and IL-2-mediated STAT5 signaling pathway. After IFN-γ activates STAT1, or IL-2 activates STAT5, AhR interacts with STAT1 and STAT5, thereby inhibiting the differentiation of Th17. Experiments by Quintana et al. showed that activated AhR regulates STAT1 but contributes to the differentiation of iTreg (96). When TGF-*β* induces iTreg differentiation, AhR can also mediate the transcription factors Smad1 and Aiolos to promote Treg differentiation. Smad1 can regulate the +2079 to +2198 sequence of the Foxp3 promoter, and Aiolos forms a complex with Foxp3 to silence the expression of IL-2, thereby increasing the expression of Foxp3 to play an immune regulatory role (97).

In terms of regulating the epigenetic level, Kim et al. found that IL17A mRNA was highly expressed in peripheral blood mononuclear cells of IBD patients, and the IL-17A promoter was hypomethylated (98). Singh et al. found that TCDD can induce the differentiation of iTreg after activating AhR, but not the differentiation of Th17 (82). There are many studies on transcription factors and cytokines that regulate Th17 differentiation, but non-coding RNAs are relatively scarce. Among them, Th17-related microRNA, namely miR-326, is related to the occurrence of autoimmune diseases such as multiple sclerosis and autoimmune encephalomyelitis. Highly expressed miR-326 can promote the differentiation of Th17 and aggravate the occurrence of disease (99). However, Gasch et al. found that the expression of miR-326 was not related to the differentiation environment of T lymphocytes, but related to the AhR ligand FICZ, which down-regulated the expression of miR-326 in CD4+ T cells, thereby inhibiting the expression of IL-17A (100).

**Figure 2:**
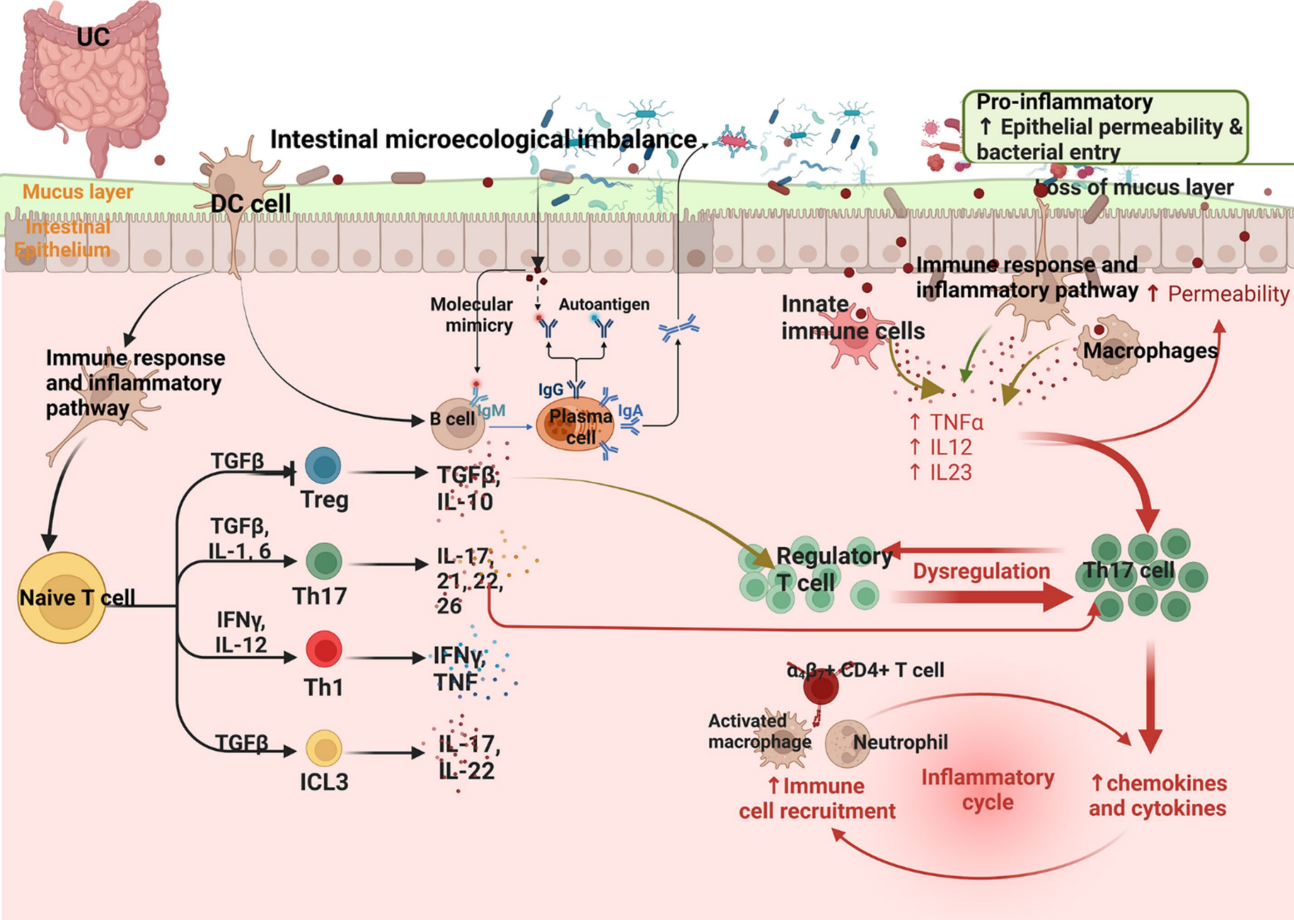
Summary of the mechanism of UC (Changes in the microbiota in UC and a reduction in the mucus layer lead to barrier breakdown, promoting access of the microbiota to the epithelial barrier, which in turn leads to infiltration of immune cells by binding to adhesion molecules expressed by the vascular endothelium. Infiltrating monocytes maturing into macrophages produce TNF, IL-12, IL-23, and IL-6, leading to Th1 cell polarization. Epithelial-derived IL-36γ suppresses Tregs and induces the polarization of IL-9-producing Th9, and IL-36 can induce fibrogenesis genes).

The mechanisms of UC are summarized in Figure 2.

## 6 Modulation of Th17/Treg balance in UC by phytochemicals

### 6.1 Natural plant ingredient monomer, component or active ingredient

**Figure 3:**
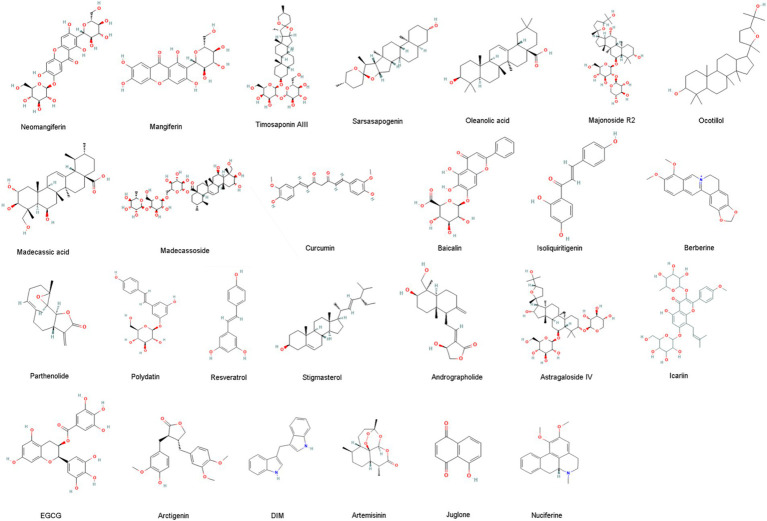
The structures of phytochemicals described in Section 6.1.

Monomers, components, or active ingredients of many natural plant active ingredients have regulatory effects on Th17/Treg balance in UC. These include extracts of various types of compounds such as glycosides, polysaccharides, volatile oils, alkaloids, flavonoids, polysaccharides, and natural plant active ingredients (Table 1; Figure 3).

#### 6.1.1 Paeoniflorin and total glycosides of paeony

TGP, derived from the dried root of *Paeonia lactiflora* Pall, encompasses active components like paeoniflorin, hydroxypaeoniflorin, and paeoniflorin. Known for its anti-inflammatory, immunomodulatory, antithrombotic, and hepatoprotective properties, TGP plays a crucial role in curbing autoimmune responses, thereby upholding immune tolerance within the body (101, 102). Lin et al. observed that TGP significantly curtails Th17-related cytokines like IL-17 and TNF-*α* in rats afflicted with UC while elevating Treg-related cytokines such as TNF-*β*, and IL-10, among others. This suggests that TGP mitigates intestinal mucosal damage in UC by orchestrating cytokine production, quelling the effector attributes of Th17 cells, and enhancing Treg responses (103). Basic research has revealed that paeoniflorin restores the balance of Th17/Treg, inhibits the activation of the NF-κB signaling pathway, reduces oxidative stress, suppresses the secretion of inflammatory factors such as IL-17, TNF-α, and ICAM, while simultaneously promoting the expression of autophagy-related factors such as LC3BII and Beclin1, thus ameliorating UC (104–106). Paeoniflorin also aids in improving TNBS-induced colitis by modulating the Th17/Treg balance mediated by DCs (107).

#### 6.1.2 Neomangiferin and mangiferin

Mangiferin and neomangiferin (Figure 3) are present in *Anemarrhena asphodeloides* and mango pistils (from the *Anacardiaceae* family), commonly utilized in herbal remedies and functional foods to address inflammation, asthma, and pain management (108–110). Mangiferin exhibits antioxidants, analgesics, anti-inflammatories, and effects on colitis and diabetes (111–113). Neomangiferin, more hydrophilic than mangiferin, benefits osteoporosis and lipid issues (110, 114). *In vitro* studies indicate that neomangiferin restores the balance of Th17/Treg cell populations by downregulating IL-17 and RORγt expression while upregulating IL-10 and FOXP3 expression, ameliorating colitis (115). Meanwhile, mangiferin modulates innate immunity by inhibiting TLR4-NF-κB/MAPK signaling, particularly IRAK1 phosphorylation, exhibiting anti-colitis effects (116). In a TNBS-induced colitis mouse model, mangiferin significantly reduced colon shortening and myeloperoxidase activity, potentially by impeding Th17 cell differentiation, decreasing TNBS-induced IL-17 expression, promoting Treg cell differentiation, and enhancing TNBS-inhibited IL-10 expression. Additionally, mangiferin inhibited spleen cell differentiation into Th17 cells *in vitro* while promoting Treg cell differentiation. This may involve downregulating RORγt and IL-17 expression, inhibiting STAT3 activation in spleen cells, upregulating Foxp3 and IL-10 expression, and enhancing STAT5 activation (117). Overall, in both *in vitro* and *in vivo* settings, mangiferin has demonstrated its ability to mitigate inflammation by modulating the differentiation of adaptive immune-related T cells.

#### 6.1.3 Timosaponin AIII and its metabolite sarsasapogenin

Timosaponin AIII (Figure 3), derived from *Anemarrhena asphodeloides* rhizomes and serving as sarsasapogenin, exhibits anti-inflammatory properties (118). Lim et al. identified that both timosaponin AIII and its metabolite sarsasapogenin effectively inhibited NF-κB and MAPK activation, as well as the phosphorylation of IRAK1, TAK1, and IκBα in LPS-stimulated macrophages. These compounds not only suppressed Th17 cell differentiation in the colonic lamina propria but also facilitated Treg cell differentiation. Additionally, timosaponin AIII and sarsasapogenin impeded the differentiation of splenic CD4+ T cells into Th17 cells *in vitro* (119).

#### 6.1.4 Oleanolic acid

Oleanolic acid (Figure 3) and its derivatives, natural pentacyclic triterpenoids extensively present in various plants, offer a range of preventive and therapeutic benefits across conditions such as UC, multiple sclerosis, and metabolic disorders (120–123). Research highlights the remarkable inhibitory prowess of a potent synthetic triterpene analog derived from oleanolic acid in interrupting cellular inflammatory pathways. Oleanolic acid showcases its inhibitory impact on induced nitric oxide synthase (iNOS) and cyclooxygenase (COX)-2 in macrophages (124). This compound also deters DSS-induced Th17 cell differentiation and lowers the expression of RORγt and IL-17 in the colon and colonic lamina propria concurrent with an upsurge in Treg cell differentiation markers, notably Foxp3 and IL-10 (125). These outcomes suggest that oleanolic acid holds promise in ameliorating inflammatory conditions like colitis by curtailing Th17 cell differentiation while enhancing Treg cell development.

#### 6.1.5 Majonoside R2 and ocotillol

The indigenous Nian hill tribes of Vietnam have traditionally harnessed the therapeutic potential of *Panax vietnamensis* Ha & Grushv., attributing it with significant anti-fatigue, life-preserving, and anti-inflammatory properties (126, 127). Notably rich in follistol saponins, particularly machonoside R2 (Figure 3), this plant showcases antistress, antidepressant, and anxiolytic effects (128). Lee et al. discovered that majonoside R2 can be metabolized into ocolitoll (Figure 3) through gut microbiota (129). Ocolitoll inhibited the TNBS-induced physiological damage, including colon shortening, increased macroscopic scores, heightened myeloperoxidase activity, and elevated production of nitric oxide and prostaglandin E2. Additionally, ocolitoll effectively suppressed Th17 cell differentiation triggered by TNBS within the colonic lamina propria, while modulating the expression levels of key markers such as T-bet, RORγt, IL-17, and IL-23. Concurrently, Treg cell differentiation was significantly enhanced, accompanied by increased expression of Foxp3 and IL-10. These findings suggest that oral administration of majonoside R2 leads to its conversion into ocolitoll through gut microbiota metabolism, thereby rebalancing the Th17/Treg ratio and offering relief in inflammatory conditions like colitis (129).

#### 6.1.6 Madecassoside and madecassic acid

*Centella asiatica* (L.) Urb. is a perennial herb acclaimed for its bioactive compounds, which include glycosides such as madecassoside (Figure 3) and asiaticoside, alongside their corresponding aglycones asiatic acid and asiatic acid (130–132). Within investigations concerning collagen-induced arthritis in rats, the madecassoside was observed to play a crucial role in rebalancing Th17/Treg cell populations (133). Remarkably, a study revealed that oral administration of madecassic acid resulted in decreased proportions of Th17 cells, accompanied by reduced expression levels of pivotal markers like RORγt, IL-17A, IL-17F, IL-21, and IL-22, while concurrently increasing Treg cell counts and enhancing the expression of Foxp3 and IL-10 in the colons of mice with colitis (134). Interestingly, under Th17 polarized conditions, madecassic acid exhibited inhibitory effects on ACC1 expression and promoted the transition of Th17 cells to Treg cells, without impacting Treg cell differentiation under Treg-polarized conditions. Overall, the conversion of madecassoside to madecassic acid elucidates a regulatory mechanism involving the PPARγ/AMPK/ACC1 pathway, aimed at restoring the Th17/Treg balance to potentially mitigate conditions such as UC (134).

#### 6.1.7 Curcumin

Curcumin (Figure 3), a naturally occurring non-toxic compound extracted from *Curcuma longa* L., possesses a myriad of biological properties, including anti-inflammatory, anti-infective, anticoagulant, and immunomodulatory actions (135, 136). Curcumin can reduce nitric oxide levels, myeloperoxidase activity, and TNF-*α*, inhibiting NF-κB activation to exert its anti-inflammatory effects, while providing protective benefits through antioxidative pathways in the treatment of DSS-induced colitis (137–139). Wei et al. observed that curcumin elevated the anti-inflammatory cytokine IL-10 while reducing pro-inflammatory cytokines like IL-23 to modulate the balance of Treg/Th17, thereby ameliorating DSS-induced colitis (140).

#### 6.1.8 Baicalin

Derived from *Scutellaria baicalensis* Georgi, baicalin (Figure 3) is a flavonoid compound deeply entrenched in TCM for its versatile therapeutic properties, including anti-inflammatory, antibacterial, anti-allergic, and anti-cancer activities (141). Recent research underscores baicalin’s profound impact on TNBS-induced colitis, showcasing remarkable reductions in disease severity indicators like disease activity index, macroscopic and microscopic scores, while simultaneously ameliorating weight loss and colon shortening (142). Intriguingly, the salutary effects of baicalin appear intertwined with the modulation of Th17 and Treg cell dynamics. Treatment with baicalin notably dampened the population of Th17 cells, along with suppressing Th17-associated cytokines (IL-17 and IL-6) and repressing RORγt expression. Moreover, baicalin demonstrated the potential to enhance Treg cell numbers, elevate Treg-linked cytokines such as TGF-*β* and IL-10, and boost the expression of FOXP3 (142).

#### 6.1.9 Isoliquiritigenin and naringinin

The primary constituents of *Glycyrrhizae radix* Et Rhizoma include Glycyrrhizin and flavonoids like liquiritigenin and isoliquiritigenin (Figure 3), along with their aglycones. Studies have shown that both *Glycyrrhizae radix* Et Rhizoma and isoliquiritigenin, a component thereof, possess the ability to impede NF-kB activation induced by LPS and the activation of the NLRP3 inflammasome (143–145). Notably, Glycyrrhizin aids in mitigating tissue inflammation by diminishing reactive oxygen species (ROS) production by neutrophils (146). In addition, Guo et al. uncovered that isoliquiritigenin and naringin facilitate the generation of Treg cells, both in laboratory settings and in live subjects. This observation suggests that the enhancement of regulatory T-cell development could serve as the fundamental mechanism behind the long-standing therapeutic efficacy of *Glycyrrhizae radix* Et Rhizoma, suggesting these two potent molecules as valuable resources in combatting autoimmune and inflammatory disorders (147).

#### 6.1.10 Berberine

Berberine (Figure 3), an alkaloid isolated from *Rhizoma Coptidis*, exhibits many biological functions and has been used in the treatment of diarrhea, gastroenteritis, diabetes, hyperlipidemia, and UC (148). Studies have shown that berberine can improve the balance of Treg/Th17 in DSS-induced UC model, and also reduce the diversity of intestinal microbiota, affecting the relative abundance of Desulfovibrio, Eubacterium and Bacteroides (149). Tang et al. found that berberine can regulate the expression of various inflammatory factors, proteins and miRNAs through the miR-31-5p-Th17/Treg immune network axis, and exert a therapeutic effect on TNBS/ethanol-induced UC (150).

#### 6.1.11 Parthenolide

Parthenolide (Figure 3), a sesquiterpene lactone extracted from *Tanacetum balsamita* branches, demonstrates robust bioactivity encompassing potent anticancer, anti-inflammatory, and antibacterial effects (151). Liu et al. reveal that mice treated with parthenolide exhibited marked improvements in colonic inflammation, accompanied by reductions in body weight, colon length, disease activity index, histological scores, and colonoscopy scores (152). Parthenolide exerts selective actions by increasing Treg cell levels while decreasing the proportion of colonic Th17 cells, thus enhancing the Treg/Th17 equilibrium crucial for intestinal stability. To validate this microbiota-dependent mechanism, experiments involving gut microbiota depletion and fecal microbiota transplantation (FMT) were conducted. The findings suggest that parthenolide substantially boosts the concentration of short-chain fatty acids in mice with colitis by regulating gut microbiota, specifically targeting short-chain fatty acid-associated bacteria such as Alloprevotella, Rikenella, and Fournierella, subsequently fostering Treg/Th17 balance (152).

#### 6.1.12 Polydatin

Polydatin (Figure 3) is a natural component extracted from the dried rhizome of *Polygonum cuspidatum Sieb. et Zucc.*, which has anti-fibrosis, anti-tumor, anti-atherosclerotic disease, anti-hepatitis, and prevention of multiple organ ischemia–reperfusion injury and other activities (153). Liu et al. found that Polydatin significantly alleviated DSS and TNBS-induced colitis in mice, and significantly reduced the proportion of Th17 cells in the spleen and mesenteric lymph nodes (154). Mechanistic studies have shown that polydatin can relieve colitis by directly binding to STAT3-specific inhibitory signal transducers and STAT3 phosphorylation activators, leading to the reduction of Th17 cells. These findings provide new insights into the anti-colitis effects of Polydatin, which may be a promising drug candidate for the treatment of IBD (154).

#### 6.1.13 Resveratrol

Resveratrol (Figure 3), a naturally derived active compound present in grapes, peanuts, and various plant sources, exhibits a diverse array of biological functions, including immunomodulation, anti-inflammatory properties, antioxidant effects, anti-angiogenic activity, and mitigation of tissue damage (155, 156). Notably, resveratrol showcases remarkable therapeutic potential in addressing UC (UC) by diminishing neutrophil exudation, thwarting adhesion molecules, and fine-tuning cytokine levels (157, 158). Clinical investigations underscore the efficacy of anti-inflammatory interventions in managing UC patients. Yao et al. found that resveratrol may restore Treg/Th17 equilibrium, elevate TGF-β1 and IL-10 concentrations, reduce IL-6 and IL-17 levels, and inhibit the hypoxia-mTOR-HIF-1α-Th17 as well as IL-6-STAT3-HIF-1α-Th17 pathways (159). Additionally, Gu et al. observed that resveratrol can rectify the Treg/Th17 immune imbalance and curb intestinal inflammatory responses in UC cases, highlighting its promising immunomodulatory properties (160).

#### 6.1.14 Dihydromyricelin

Dihydromyricelin is a dihydroflavonol flavonoid compound, which has various pharmacological effects such as anti-oxidation, anti-tumor, anti-inflammation, anti-alcohol and liver protection, anti-pathogenic microorganisms and blood lipid regulation (161). Li et al. found that Dihydromyricelin may restore the balance of Treg/Th17 in peripheral blood and reduce the expression of MMP9 in colon tissue, so as to alleviate DSS-induced UC in mice (162).

#### 6.1.15 Daphnetine

Derived from daphne coumadin, DAPH is a coumarin derivative renowned for its robust anti-inflammatory and antioxidant characteristics, commonly employed in combatting inflammatory ailments (163–165). DAPH demonstrates the capacity to impede T h17 cell differentiation while fostering Treg cell maturation, thereby fostering immune equilibrium and ameliorating inflammation (166, 167). Previous research indicates that DAPH can shield against intestinal inflammatory disorders in a rat model of TNBS-induced colitis, with its intestinal anti-inflammatory efficacy linked to its antioxidant profile (165). A novel investigation reveals that this compound substantially boosts the population of gut microbiota capable of producing short-chain fatty acids (SCFAs), a shift associated with bolstering Treg development and diminishing the differentiation of pro-inflammatory T cells into h17 cells, ultimately quelling UC (168).

#### 6.1.16 Stigmasterol

Stigmasterol (Figure 3), a phytosterol renowned for its anti-inflammatory, antioxidant, and antitumor attributes, and its positive impacts on metabolism (169, 170), serves as a key active component in traditional Chinese herbal mixtures utilized for managing inflammatory bowel disease (IBD) (171, 172). Prior investigations unveiled stigmasterol’s capacity to notably diminish cyclooxygenase-2 (COX-2) expression levels, leading to enhanced colonic inflammation scores and amelioration of colitis symptoms. Treatment with stigmasterol was shown to rebalance the Treg/Th17 equilibrium and induce alterations in gut microbiota composition in a colitis model induced by DSS. Moreover, stigmasterol treatment amplified the generation of short-chain fatty acids (SCFAs) by gut microbiota, particularly boosting butyrate production. Researchers hypothesized that stigmasterol might rectify the Treg/Th17 cell imbalance by activating PPAR*γ* via butyrate, thus offering therapeutic benefits in addressing IBD concerns (173).

#### 6.1.17 Citrus flavonoids

Citrus flavonoids, like psilocybin and tangeretin, are naturally occurring compounds found abundantly in the rinds of citrus fruits, particularly *Citrus* species (174). These compounds boast a spectrum of biological activities encompassing anti-inflammatory, anticancer, hypolipidemic, anti-obesity, and neuroprotective effects (175–179). They demonstrate efficacy in ameliorating dermal responses by mitigating histamine effects and triggering the activation of transcription factors NF-κB and AP-1 via protein kinase C pathways (180, 181). In UC, tangeretin effectively suppresses TNF-*α*, IL-12, and IL-23 expression, alongside NF-κB activation in lipopolysaccharide-stimulated dendritic cells. Moreover, Tangeretin impedes the differentiation of Th1 and Th17 cells induced by TNBS, restraining the expression of T-bet, ROR*γ*t, interferon-γ, IL-12, IL-17, and TNF-α, while concurrently promoting the differentiation of Treg cells suppressed by TNBS, upregulating Foxp3 and IL-10 expression levels (182).

#### 6.1.18 Andrographolide

Andrographolide (Figure 3), an therapeutic agent with clinical efficacy in treating bronchitis, tonsillitis, and bacillary dysentery (183, 184), has garnered attention in recent research for its ability to modulate macrophage activation, suppress Th1/Th17 immune responses, and diminish MAPK and NF-κB signaling pathways, beneficial for managing conditions like acute colitis and lung injuries (185, 186). Noteworthy findings indicate that andrographolide sulfonate notably ameliorated TNBS-induced symptoms including weight loss, myeloperoxidase activity, colon shortening, and colonic inflammation, while reducing the expression of pro-inflammatory cytokines at both mRNA and protein levels. Moreover, andrographolide sulfonate impeded the infiltration of CD4+ T cells and the differentiation of Th1 (CD4+ IFN-γ+) and Th17 (CD4+ IL17A+) subsets. Studies illustrated that andrographolide sulfonate successfully mitigated TNBS-induced colitis in mice through the inhibition of Th1/Th17 immune responses (187).

#### 6.1.19 Astragaloside IV and Astragalus polysaccharide

Astragaloside IV (Figure 3), a triterpene saponin derived from Astragalus membranaceus (Fisch.) Bge., is renowned for its wide-ranging beneficial effects, spanning antioxidant, cardioprotective, anti-inflammatory, antiviral, antibacterial, antifibrotic, antidiabetic, and immunomodulatory properties (188). Notably, in models of experimental colitis triggered by DSS and TNBS, astragaloside IV demonstrated the ability to modulate macrophage polarization via the STAT signaling pathway and influence energy metabolism, thereby effectively mitigating intestinal inflammation (189, 190). Furthermore, astragaloside IV exhibited the capacity to lower levels of key pro-inflammatory cytokines such as myeloperoxidase, TNF-*α*, IL-1β, IL-6, and nitric oxide *in vitro* (191). Recent research findings underscored that astragaloside IV not only ameliorated clinical symptoms associated with DSS-induced colitis but also significantly enhanced ulcer repair, reduced inflammatory cell infiltration and inflammation indices, and regulated the expression of inflammatory cytokines within colon tissues. Of significant note, early administration of astragaloside IV was found to restore Th17/Treg cell balance in mice with acute colitis, inhibiting Th17 responses while promoting Treg cell responses in cases of recurrent colitis (192, 193).

Astragalus polysaccharide, the primary bioactive compound derived from Astragalus membranaceus (Fisch.) Bge, showcases a diverse array of biological properties encompassing antiviral, antitumor, anti-aging, anti-radiation, anti-stress, and antioxidant activities (194, 195). In the context of DSS-induced colitis, Astragalus polysaccharides exhibited therapeutic effects by impeding the NF-κB and NRF2/HO-1 signaling pathways (196, 197). Recent investigations unveiled that these polysaccharides enhanced the survival rates, disease activity indices, body weight fluctuations, colon lengths, body weights, and mitigated colonic histopathological damage in a mouse model of UC. Notably, Astragalus polysaccharides orchestrated the expression levels of inflammatory cytokines (IL-2, IL-6, IL-12p70, IL-23, TNF-ɑ, and TGF-β1) in the colon tissues of colitis-afflicted mice. Moreover, Astragalus polysaccharide significantly upregulated Tfh10 and Tfr populations while downregulating Tfh1, Tfh17, and Tfh21. Additionally, these polysaccharides elevated Treg cell levels along with pertinent nuclear transcription factor Foxp3 and cytokine IL-10 expression in colitis models. In essence, Astragalus polysaccharide holds promise in alleviating UC by rebalancing the Tfh/Treg cell equilibria (198).

#### 6.1.20 Icariin

Derived from Epimedium brevicornu Maxim, icariin (Figure 3) is a natural flavonoid glucoside recognized for its diverse pharmacological attributes (199), spanning antioxidant, antidepressant, and anti-inflammatory properties (200, 201). Oral intake of icariin markedly decelerates disease advancement and mitigates pathological alterations in colitis conditions, and effectively curbs the generation of pro-inflammatory cytokines and the activation of p-p65, p-STAT1, and p-STAT3 within colon tissues (202). Further investigations unveiled the dose-dependent capacity of icariin to impede the proliferation and activation of T lymphocytes, along with lowering pro-inflammatory cytokine levels in stimulated T cells. Moreover, icariin administration was shown to suppress the phosphorylation of STAT1 and STAT3, pivotal transcription factors associated with Th1 and Th17 responses, within CD4+ T cells (202). Collectively, these outcomes underscore the potential of icariin as a viable therapeutic option for addressing IBD.

#### 6.1.21 Epigallocatechin-3-gallate

Hailing from green tea, EGCG (Figure 3) stands as a natural compound with a spectrum of beneficial attributes including antibacterial, anti-inflammatory, antitumor, antioxidant, and anti-aging properties (203, 204). Notably, research highlights its capacity to modulate the immune system, showcasing potential in the realm of autoimmune disease treatment (205, 206). EGCG interventions led to decreased IL-6 and IL-17 release, alongside the adjustment of Treg/Th17 ratios within mouse spleens. Concurrently, elevated plasma levels of IL-10 and TGF-β1 were observed, while the expression of HIF-1α and STAT3 proteins in the colon was curtailed. These findings hint at EGCG’s potential in ameliorating experimental colitis in mice by stifling IL-6 release and orchestrating the Treg/Th17 equilibrium *in vivo* (39).

#### 6.1.22 Arctigenin

Within the realm of herbal medicine lies Arctigenin (Figure 3), a bioactive dibenzylbutyrolactone lignan renowned for its array of properties. Its repertoire spans antimicrobial, anti-inflammatory, and immune-modulating capabilities, with a recent surge of interest stemming from its antitumor prowess (20, 207, 208). Deemed an ERβ agonist in a recent investigation, Arctigenin exhibits a moderate affinity for ERβ, initiating the dissociation of the ERβ/HSP90 complex, enhancing ERβ phosphorylation for nuclear translocation, and elevating transcriptional activity levels. Through ERβ activation, Arctic Aglycon impedes mTORC1 function by disrupting ERβ-constrained-mTOR complex interaction. Intriguingly, the absence of ERβ effectively annulled Arctigenin-induced suppression of Th17 cell differentiation, hinting at ERβ as a prime candidate for Arctigenin’s target protein. This regulatory mechanism obstructs mTORC1 activation, hence thwarting Th17 cell differentiation and the progression of colitis (209).

#### 6.1.23 3,3′-Diindolylmethane

Emerging from the breakdown of glucosinolate in cruciferous vegetables, DIM is a natural compound known for inhibiting inflammatory responses in select mouse inflammation models (Figure 3) (210, 211). Recent investigations have spotlighted DIM’s protective role in inflammation mitigation, showcased by its ability to curb NF-κB activation, stifle prostaglandin E2 production, and modulate antioxidant levels (212, 213). A novel study underscored how DIM administration assuaged experimental colitis, suggesting its influence on the downstream signaling pathways of AhR could reduce Th2/Th17 cells while bolstering Treg populations. Demonstrating therapeutic efficacy in animal models of UC, DIM’s capability to suppress Th2/Th17 cells and boost Tregs positions it as a promising therapeutic avenue for UC patients (214).

#### 6.1.24 Artemisinin

Artemisinins (Figure 3), derived from *Artemisia annua* L., encompass a collection of sesquiterpene trioxanes lauded for their therapeutic potential (215, 216). Dihydroartemisinin (DHA), an artemisinin derivative, emerges as a robust immunomodulator with diverse applications. Studies indicate the efficacy of DHA and its derivatives in managing autoimmune conditions, such as experimental autoimmune encephalomyelitis (EAE) (217) and collagen-induced arthritis (218). Yan et al. discovered that DHA inhibited Th1, Th17, Th9, and Th22 cell populations in TNBS- or OXA-induced colitis, while enhancing Tregs (219). Remarkably, DHA induced HO-1 production, promoted CD4+ T cell apoptosis, and restored the Th17/Treg balance, effects that were blocked by the HO-1 inhibitor Sn-protoporphyrin IX. Overall, these results suggest that DHA has the potential to serve as a novel therapeutic agent for managing inflammatory bowel disease (IBD) or Th17/Treg immune modulation (219).

#### 6.1.25 Juglone

Derived from the green walnut shell of *Juglans regia* L., juglone (Figure 3) stands out as a pivotal naphthoquinone compound recognized for its manifold properties (220, 221). Recent investigations have highlighted juglone’s anti-inflammatory prowess, showcasing efficacy in conditions like hepatitis, neuroinflammation, and allergic pulmonary fibrosis (222, 223). Juglone has been shown to improve the disease activity index and pathological features of UC, significantly reducing the protein levels of IL-6, TNF-*α*, and IL-1β, while enhancing IL-10 expression (224). Moreover, Juglone induces changes in microbial diversity and gut microbiota composition, promoting the ratio of Firmicutes to Bacteroidetes and the abundance of Actinobacteria, while inhibiting the levels of Proteobacteria (224). This compound also inhibits the protein levels of IL-6, STAT3, and RORγt, thereby enhancing FOXP3 expression. Additionally, Juglone impedes the progression of Th17 cells and promotes the generation of Tregs, essential for maintaining the Th17/Treg balance (224). These findings suggest that Juglone can protect mice from UC by shaping the dynamics of gut microbiota and restoring the balance of Th17/Treg.

#### 6.1.26 Nuciferine

*Nelumbo nucifera* Gaertn. is used in TCM for fever, diarrhea and bleeding (225). Nuciferine, an alkaloid containing an aromatic ring, is the main bioactive component derived from *Nelumbo nucifera* Gaertn. (226). Studies reveal nuciferine’s potential in counteracting high-fat diet-induced obesity in mice through gut microbiota modulation (227, 228), mitigating inflammation triggered by fructose or uric acid in HK-2 cells, among other beneficial effects (229). In research, nuciferine showcased its efficacy in alleviating symptoms in DSS-induced UC mice, such as mitigating histological damage and colon shortening. Notably, nuciferine played a role in enhancing the Th1/Th2 and Treg/Th17 equilibrium, alongside influencing gut microbiota composition within DSS-induced inflammatory bowel disease (IBD) models (230).

### 6.2 Traditional Chinese medicine prescriptions

In this section, different TCM prescriptions (Table 2) for treatment of UC will be discussed.

#### 6.2.1 Kuijieling

KJL, a TCM formulation deeply rooted in empirical practices, addresses UC by employing a therapeutic approach that involves heat dissipation, spleen fortification, and blood circulation enhancement (231). Noteworthy outcomes in clinical assessments underscore its effectiveness (232). Experimental investigations reveal that KJL can mitigate ulceration, ameliorate pathological tissue damage, and diminish levels of inflammatory markers such as IL-1β, TNF-*α*, and IL-6 in UC-induced rats (232–234). KJL enhances the levels of blood TGF-β1, IL-2, IL-10, increases colonic Foxp3, STAT5, and IL-2 levels, while decreasing the levels of IL-6, IL-23, IL-17, IL-21 in the blood and inhibiting colonic-related RORγt, STAT3, IL-23, IL-17, and IL-21, thereby restoring the balance of Th17/Treg (235, 236).

#### 6.2.2 Baitouweng Tang

BTWT, a prevalent TCM concoction majorly compounded with pulsatilla, has gained substantial traction in UC therapy across China (237–239). Tan et al. discovered that BTWT could downregulate RORγt expression, upregulate Foxp3 expression, increase the proportion of Treg cells in patients, decrease the proportion of Th17 cells, thereby restoring the Th17/Treg balance in the body (240).

#### 6.2.3 Fuzi Lizhong Tang

With a historical backdrop spanning millennia, FZLZT stands as a stalwart remedy against gastrointestinal maladies, encompassing conditions such as enteritis, diarrhea, and gastritis (241–243). Shedding light on its therapeutic potential, Li et al. unveiled FZLZT’s capacity to ameliorate UC symptoms linked to spleen-kidney yang deficiency, attenuate colonic tissue inflammation in rat models, and facilitate mucosal regeneration within the colon (244). Furthermore, FZZLT bolsters the expression of Treg-associated factors like IL-10, TGF-β1, Foxp3, and STAT5, thereby contributing significantly to the management of UC characterized by spleen-kidney yang deficiency (244).

#### 6.2.4 Qingchang Wenzhong Tang

QCWZT has showcased clinical efficacy in alleviating symptoms of UC and has obtained authorization for further clinical investigations. Research findings underscore its notable capacity to effectively suppress the active phase of UC, exhibiting comparable outcomes to mesalamine (245). Moreover, QCWZT demonstrates the ability to diminish the modified Mayo score in patients grappling with mild to moderate UC, enhance pathological repair, curtail inflammatory cellular infiltration in the intestinal mucosa, promote colonic goblet cell proliferation, elevate mucin MUC2 expression in colonic mucosal tissue, and impede STAT6 expression (245). In a parallel discovery, Sun et al. proposed that QCWZT may modulate the VDR signaling pathway by downregulating miR-675-5p expression, thereby harmonizing the Th17/Treg immune equilibrium in UC, remedying intestinal mucosal barrier impairments, and aiding in UC management (246). Recent investigations have indicated that the bioactive constituents of QCWZT possess the capability to suppress the IL-6-STAT3 pathway, ultimately impeding the differentiation of Th17 lymphocytes, thereby diminishing colonic inflammation (247).

#### 6.2.5 Fufang Kushen Tang

Clinical evaluations have affirmed the therapeutic efficacy of FFKST in managing UC, showcasing a capacity to diminish intestinal mucosal inflammation in afflicted individuals. This herbal formulation has proven instrumental in alleviating abdominal pain and hematochezia symptoms (248). Primarily constituted of active components like matrine, gallic acid, indigo, notoginsenoside R1, ginsenoside Rb1, and glycyrrhizic acid, FFKST has demonstrated inhibitory properties at a molecular level across various UC models. Matrine, for instance, mitigated colitis manifestations and curbed inflammatory cytokine expression of IFN-*γ* and IL-17 in TNBS-induced colitis within IL-10-deficient mice (249). Oxymatrine, when tested in a UC model, ameliorated intestinal damage by modulating T cell-secreted inflammatory cytokines and inhibiting NF-κB activation (250, 251). Moreover, researchers noted significant enhancements in colitis symptoms and pathological mitigation in mice treated with FFKST, alongside notable effects on immune modulation through the regulation of Th17/Treg cell balance in DSS-induced colitis models (252, 253).

#### 6.2.6 Dahuang Mudan Tang

Originally documented in the ancient text *Jingui Yaolüe*, DHMDT emerges as a viable solution for intestinal abscesses (254). Discoveries by Luo et al. unveiled the remarkable efficacy of DHMDT in mitigating pathological alterations in UC-afflicted mice. This included the restoration of colon length, amelioration of weight loss, and tissue repair in conjunction with inflammation reduction. Additionally, DHMDT precipitated a shift in gut microbiota composition, enhancing alpha diversity while notably elevating Firmicutes and Actinobacteria populations and diminishing Proteobacteria and Bacteroidetes numbers. Noteworthy was the substantial augmentation of butyrate-producing Butyricoccus elongatum colonies and the restoration of short-chain fatty acid levels in the gut. DHMDT also fostered an improved Th17 cell/Treg cell ratio in mesenteric lymph nodes and a decline in various inflammatory cytokines like IL-6, TNF-*α*, IFNγ, IL-10, IL-17A, IL-21, and IL-22 within the colon (255).

#### 6.2.7 Gegen Qinlian Tang

Utilized clinically for acute enteritis, chronic diarrhea, and bacillary dysentery, GQT harbors a diverse array of potent bioactive compounds like berberine, baicalin, and puerarin. Recent studies suggest that these constituents possess the ability to modulate T cell differentiation individually, offering promise in mitigating T cell-associated inflammatory disorders across various animal models (256–258). Hu et al. reported significant symptom relief in UC-afflicted mice following GQT treatment, alongside noteworthy inhibition of myeloperoxidase activity. Notably, the administration of GQT led to a substantial decrease in pro-inflammatory factors like IL-1β, TNF-α, and IL-6. This herbal formulation facilitated the infiltration of Treg and Th17 cells into the colon while concurrently reducing the expression of inflammatory mediators such as TGF-β1 and IL-17. Through the suppression of IL-6/JAK2/STAT3 signaling, GQT is postulated to restore Treg and Th17 cell equilibrium within colon tissue, ultimately easing DSS-induced UC symptoms (259). Wang et al. additionally observed that modified GQT modulates the Treg/Th17 balance and effectively addresses DSS-triggered acute experimental colitis in mice by reshaping the gut microbiota composition (260).

#### 6.2.8 Shenling Baizhu san

In a study by Tian et al., it was observed that when SLBZS was administered in conjunction with standard therapy, patients exhibited significantly lower rates of colonoscopic congestion, edema, erosion, and ulcers compared to those in the control group. This indicates the potential benefits of this combined approach in aiding the restoration of intestinal mucosal integrity in individuals with UC. The purported efficacy of SLBZS on mucosal repair appears connected to its capability to address Th17/Treg imbalances and temper inflammatory responses in UC patients. Notably, the post-treatment recurrence rate in the observation group was notably lower than in the control group, underscoring the potential role of SLBZS in rectifying immune irregularities among patients (261). The therapeutic mechanisms of SLBZS in UC management could entail modulating the expression levels of RORγt/FoxP3 and rectifying the Th17/Treg immune imbalance. Furthermore, Qi et al. highlighted that SLBZS’s protective influence in UC rats might also be linked to adjusting the RORγt/FoxP3 expression levels and correcting Th17/Treg immune discrepancies (262).

#### 6.2.9 *Panax ginseng* C. A. Mey. extracts

Extracts from *Panax ginseng* C. A. Mey. contain essential components like ginsenosides Rb1, Rb2, Re, and Rg1, utilized in TCM to address conditions such as inflammation, cancer, and diabetes (263, 264). These ginsenosides are known for their anti-inflammatory properties and immune-regulating abilities (265–267). For instance, ginsenoside Rb1 undergoes gut microbiota metabolism to produce 20-O-(*β*-D-glucopyranosyl)-20(S)-protopanaxadiol (compound K), which has demonstrated suppressive effects on LPS-induced inflammation by targeting IRAK1 in macrophages and enhancing outcomes in TNBS-induced colitis models in mice (268).

In the realm of ginsenoside research, ginsenoside Re, belonging to the prototalol-type ginsenosides, was found effective in ameliorating TNBS-induced colitis by impeding the LPS-TLR4 binding on macrophages, thus showcasing its potential as an anti-colitis agent (249). Recent investigations unveiled that oral administration of ginsenosides Rg1, Rh1, or 20(S)-protopanaxadiol could counter TNBS-induced colon narrowing, suppress myeloperoxidase activity, and alleviate the expression of pro-inflammatory cytokines like IL-1β, IL-17, and TNFα. Additionally, these compounds exhibited the capability to hinder NF-κB activation, restore the Th17/Treg balance disrupted by TNBS, and revive the expression of key regulatory molecules like IL-10 and Foxp3. *In vitro* studies indicated their ability to inhibit Th17 cell differentiation, with 20(S)-protopanaxadiol showcasing the most potent anti-inflammatory effects, followed by Rh1. The transformation of ginsenoside Rg1 into 20(S)-protopanaxadiol by ginsenoside Rh1 and F1 unveiled a potential route for therapeutic benefits (269).

These metabolites, particularly 20(S)-protopanaxadiol, were found effective in ameliorating inflammatory conditions like colitis by impeding LPS-TLR4 interaction on macrophages and restoring Th17/Treg balance. Long et al. further highlighted the potential of ginsenoside Rg1 in mitigating DSS-induced UC in mice by modulating the Treg/Th9 cell equilibrium (270).

#### 6.2.10 Shaoyao Tang

Originating from the Jin and Yuan Dynasties, SYT represents a traditional Chinese medicinal formula known for its therapeutic benefits in managing UC (271). Recent investigations have shed light on its potential mechanisms, including the regulation of immune factors within immune cells, inflammation inhibition, and mitigation of oxidative stress (272–274). In a study conducted by Lu et al., it was observed that SYT led to a significant reduction in serum IL-17 levels and an increase in serum IFN-*γ* and IL-27 levels among rats afflicted with gastroenteric fever-type UC. This suggests that the therapeutic efficacy of SYT could be attributed to the modulation of serum IL-17 levels alongside elevated concentrations of IL-27 and IFN-γ (275).

Our previous research has highlighted the effectiveness of SYT in UC treatment, indicating a possible mechanism of action linked to the restoration of Treg/Th17 balance through the inhibition of HIF-1α (22). Furthermore, findings by Yao et al. unveiled that SYT exhibited significant relief of intestinal symptoms in UC patients characterized by damp-heat syndrome in the large intestine. By fostering intestinal mucosa recovery and enhancing patient quality of life, SYT was shown to regulate the Th17/Treg cell balance effectively (276).

### 6.3 Limitations of the current study

Although the research on natural plant components mentioned above has demonstrated potential advantages in treating UC, there are still some shortcomings.

(1) Most studies are animal or cell experiments lacking clinical research. While animal studies can provide valuable insights into potential therapeutic effects, they may not always directly apply to humans as the efficacy and safety observed in animal models may not be replicated in clinical trials involving human participants.
(2) Inconsistent handling of compounds in different studies. Due to factors such as plant variability, processing methods, and storage conditions, the components, potency, and quality of traditional herbal medicines may vary. The lack of standardization and quality control measures may lead to inconsistent treatment outcomes and potential safety issues.
(3) Compared to single-agent drugs, some traditional plant therapies (e.g., TCM prescriptions) lack rigorous scientific validation and may have side effects as well as interactions with other medications or health conditions. Without comprehensive clinical trials and post-market monitoring, identifying and managing potential risks may be challenging.
(4) Integrating traditional medicine into modern healthcare systems may pose challenges in cultural, ethical, and regulatory aspects. Concerns may arise regarding cultural appropriation, sustainability of plant resources, and equitable access to healthcare opportunities.

In addressing the above limitations, future research could involve more clinical trials (e.g., randomized controlled trials) focusing on natural plant components (including plant extracts and TCM prescriptions), comprehensively studying their mechanisms of action and safety, exploring ways to integrate them into modern healthcare systems to establish safety, efficacy, and optimal dosing regimens. Additionally, future researchers should emphasize quality control and standardization of natural compounds to ensure reproducibility of efficacy.

## 7 Prospect

In summary, IBD is a common disorder associated with a shift in the Th17/Treg balance towards a pro-inflammatory Th17 program, which negatively affects quality of life. Th17/Treg, as two closely related aspects of the immune responses, is crucial in maintaining the body’s immune homeostasis. A large amount of clinical and experimental evidence clearly shows that the regulation of Th17/Treg is an important mechanism of action of natural plant ingredients in the treatment of UC, such as the regulation of Th17/Treg regulation of transcription factor differentiation (TRORγt, STAT3, Foxp3 and STAT5), and regulate Th17/Treg signaling pathways (mTOR signaling pathway, PI3K/ Akt signaling pathway, AMPK signaling pathway, Nrf-2/HO-1 signaling pathway, HIF-1α signaling pathway, Notch signaling pathway, Wnt signaling pathway). This review mainly summarizes and analyzes the previous studies on the regulation of Th17/Treg balance in UC by natural plant components and TCM compounds and provides references for clinical rational design and treatment of UC. After summarizing, it was found that glycosides, polysaccharides, volatile oils, alkaloids, flavonoids, polysaccharides, and other types of compounds in TCM and some TCM extracts have unique regulatory advantages in regulating Th17/Treg in UC.

Currently, the impact of TCM on Th17/Treg balance in UC is underscored through several key aspects: Firstly, it works to reduce the proportion of Th17 cells, suppressing the secretion and expression of pro-inflammatory markers to alleviate intestinal inflammation and facilitate mucosal repair. Secondly, TCM aids in augmenting Treg production and differentiation, bolstering the population of anti-inflammatory cells and enhancing the secretion of related factors to reinforce intestinal immune responses. Thirdly, there is a concerted effort to diminish the presence of Th17 pro-inflammatory factors while concurrently boosting Treg anti-inflammatory factors. This synchronized modulation aims to restore balance in the Th17/Treg equilibrium, safeguarding the intestinal mucosa by reinstating immune tolerance and normal response patterns. TCM is renowned for its multifaceted, targeted effects, exhibiting minimal toxicity and side effects, thus harboring immense research and developmental value. Nevertheless, the intricate composition of TCM and its compounds presents challenges, as the precise mechanisms of action remain inadequately understood. Currently, the intricate relationship between Th17/Treg equilibrium, UC pathology, and the interventions of TCM remains elusive. Consequently, further in-depth investigations are imperative to elucidate the precise mechanisms through which TCM modulates Th17/Treg balance in UC. Advancing this understanding is essential for propelling the utilization and efficacy of TCM in addressing UC and other challenging ailments.
