# Supplementary material for: T helper cell 17/regulatory T cell balance regulates ulcerative colitis and the therapeutic role of natural plant components: a review
Source: Front Med (Lausanne). 2025 Mar 24;11:1502849. doi: 10.3389/fmed.2024.1502849 (PMC11973383; doi:10.3389/fmed.2024.1502849)
Supplement: Supplementary file 1 [file Table_1.docx]

**Table S1.** Search Strategies for Pubmed and Embase

| **PubMed** | ((Natural plant component OR Natural component OR natural compound OR Total glycosides of paeony OR Paeoniflorin OR Neomangiferin OR Mangiferin OR Temosaponin AIII OR Sarsasapogenin OR Oleanolic acid OR Ocotillol OR Majonoside R2 OR Madecassoside OR Madecassic acid OR Curcumin OR Baicalin OR Isoliquiritigenin OR Naringinin OR Berberine OR Parthenolide OR Polydatin OR Resveratrol OR Dihydromyricelin OR Daphnetine OR Stigmasterol OR Citrus nobiletin OR Andrographolide OR Astragaloside IV OR Astragalus polysaccharide OR Icariin OR Epigallocatechin-3-gallate OR Arctigenin OR 3,3'-Diindolylmethane OR Artemisinin OR Juglone OR Nuciferine) OR (traditional Chinese medicine OR Chinese medicine))  AND  (Colitis, Ulcerative OR Idiopathic Proctocolitis OR Ulcerative Colitis OR Colitis Gravis OR Inflammatory Bowel Disease, Ulcerative Colitis Type) |
| --- | --- |
| **EMBASE** | 1 Natural plant component/  2 Natural component/  3 natural compound/  4 Total glycosides of paeony/  5 Paeoniflorin/  6 Neomangiferin/  7 Mangiferin/  8 Temosaponin AIII/  9 Sarsasapogenin/  10 Oleanolic acid/  11 Ocotillol/  12 Majonoside R2/  13 Madecassoside/  14 Madecassic acid/  15 Curcumin/  16 Baicalin/  17 Isoliquiritigenin/  18 Naringinin/  19 Berberine/  20 Parthenolide/  21 Polydatin/  22 Resveratrol/  23 Dihydromyricelin/  24 Daphnetine/  25 Stigmasterol/  26 Citrus nobiletin/  27 Andrographolide/  28 Astragaloside IV/  29 Astragalus polysaccharide/  30 Icariin/  31 Epigallocatechin-3-gallate/  32 Arctigenin/  33 3,3'-Diindolylmethane/  34 Artemisinin/  35 Juglone/  36 Nuciferine/  37 1-36/or  38 traditional Chinese medicine/  39 Chinese medicine/  40 38 and 39  41 Colitis, Ulcerative/  42 Idiopathic Proctocolitis/  43 Ulcerative Colitis/  44 Colitis Gravis/  45 Inflammatory Bowel Disease, Ulcerative Colitis Type/  46 41-45/or  47 37 and 40  48 46 and 47 |
